# Co-Culture of Neural Crest Stem Cells (NCSC) and Insulin Producing Beta-TC6 Cells Results in Cadherin Junctions and Protection against Cytokine-Induced Beta-Cell Death

**DOI:** 10.1371/journal.pone.0061828

**Published:** 2013-04-17

**Authors:** Anongnad Ngamjariyawat, Kyril Turpaev, Svitlana Vasylovska, Elena N. Kozlova, Nils Welsh

**Affiliations:** 1 Department of Neuroscience, Uppsala University, Uppsala, Sweden; 2 Department of Medical Cell Biology, Uppsala University, Uppsala, Sweden, and Science For Life Laboratory (SciLifeLab), Uppsala University, Uppsala, Sweden; 3 Center for Theoretical Problems of Physicochemical Pharmacology Russian Academy of Sciences, Moscow, Russia; Sun Yat-sen University Cancer Center, China

## Abstract

**Purpose:**

Transplantation of pancreatic islets to Type 1 diabetes patients is hampered by inflammatory reactions at the transplantation site leading to dysfunction and death of insulin producing beta-cells. Recently we have shown that co-transplantation of neural crest stem cells (NCSCs) together with the islet cells improves transplantation outcome. The aim of the present investigation was to describe *in vitro* interactions between NCSCs and insulin producing beta-TC6 cells that may mediate protection against cytokine-induced beta-cell death.

**Procedures:**

Beta-TC6 and NCSC cells were cultured either alone or together, and either with or without cell culture inserts. The cultures were then exposed to the pro-inflammatory cytokines IL-1β and IFN-γ for 48 hours followed by analysis of cell death rates (flow cytometry), nitrite production (Griess reagent), protein localization (immunofluorescence) and protein phosphorylation (flow cytometry).

**Results:**

We observed that beta-TC6 cells co-cultured with NCSCs were protected against cytokine-induced cell death, but not when separated by cell culture inserts. This occurred in parallel with (i) augmented production of nitrite from beta-TC6 cells, indicating that increased cell survival allows a sustained production of nitric oxide; (ii) NCSC-derived laminin production; (iii) decreased phospho-FAK staining in beta-TC6 cell focal adhesions, and (iv) decreased beta-TC6 cell phosphorylation of ERK(T202/Y204), FAK(Y397) and FAK(Y576). Furthermore, co-culture also resulted in cadherin and beta-catenin accumulations at the NCSC/beta-TC6 cell junctions. Finally, the gap junction inhibitor carbenoxolone did not affect cytokine-induced beta-cell death during co-culture with NCSCs.

**Conclusion:**

In summary, direct contacts, but not soluble factors, promote improved beta-TC6 viability when co-cultured with NCSCs. We hypothesize that cadherin junctions between NCSC and beta-TC6 cells promote powerful signals that maintain beta-cell survival even though ERK and FAK signaling are suppressed. It may be that future strategies to improve islet transplantation outcome may benefit from attempts to increase beta-cell cadherin junctions to neighboring cells.

## Introduction

Type 1 diabetes is an autoimmune disease that results in destruction of the insulin-producing beta-cells. Cytokines, such as IL-1β, TNF-α and IFN-γ, induce beta-cell death *in vitro*; and the local release of the same cytokines has been proposed to participate in pancreatic beta-cell destruction in vivo [1]. Indeed, levels of proinflammatory cytokines have been correlated to insulitis and beta-cell destruction in both NOD mice [2] and human pancreatic biopsies from patients with recent-onset type 1 diabetes [3]. After receptor activation, cytokine-induced signaling involves the activation of mitogen-activated protein kinases (MAPK), c-Jun NH2-terminal kinase (JNK), extracellular signal-regulated kinase (ERK), and p38 MAPK [4], [5]. In addition to the mitogen-activated protein kinases, IL-1β and TNF-α–induced signaling results in activation of the pro-inflammatory transcription factor NF-κB [6]. It has been suggested that in rodent islets, cytokine-induced cell death is caused by increased nitric oxide production, which results from activation of NF-κB–mediated inducible nitric oxide synthase gene transcription [6], [7].

Transplantation of isolated islets has become an option to treat selected patients with Type 1 diabetes. However, adequate graft function is seen in less than 10% of patients after 5 years [8]. Immediate post-transplantation cell death and graft failure are likely due to hypoxia in combination with inflammatory events [9]. To increase beta-cell mass and dampen postgrafting inflammation are thus important goals for successful islet transplantation. It is possible that co-transplantation of islet cells with neural crest stem cells (NCSCs) will promote an improved islet transplantation outcome. Indeed, recent studies have demonstrated that NCSCs have an important role in beta-cell differentiation by regulating beta-cell mass during development [10]. It has also been observed that embryonic dorsal root ganglia affect insulin secretion in co-cultured islets [11], that co-cultured islets and NCSCs have mutual beneficial effects *in vitro* [12], and that co-transplantation of NCSC-derived neurospheres with islets induces proliferation and promotes function of transplanted beta-cells [13]. More recently, co-culture of insulin producing cells and NCSC resulted both in protection against cytokine-induced beta-cell death [14] and increased growth of the murine beta cell mass [15]. However, the mechanisms by which NCSCs mediate positive effect on beta cells have not yet been elucidated. Thus, the aim of the present investigation was to closer study interactions between NCSCs and insulin producing cells *in vitro*, and to correlate such interactions with improved survival in response to cytokine exposure. An understanding of such events is crucial for development of new protocols for improved outcome of islet transplantation to diabetes patients.

## Materials and Methods

### Mice

Transgenic heterozygous C57BL/6-β-actin enhanced green fluorescent protein (EGFP) mice (Jackson Laboratories, Bar Harbor, ME, http://www.jax.org) were used to generate NCSCs.

### Ethic statement

All procedures were approved by the Regional Ethics Committee for Research on Animals (The Uppsala County Regional Ethics Committee for Research on Animals).

### Beta-TC6 cells

Mouse beta-TC6 cells, purchased from ATCC, were maintained in RPMI-1640 (Gibco, Grand Island, NY, USA) supplemented with 10% fetal calf serum (Sigma Chemicals St Louis, MO, USA), 2 mM L-glutamine, streptomycin (0.1 mg/ml) and benzylpenicillin (100 U/ml). All cells were grown at 37°C in a humidified air incubator with 5% CO_2_.

### Preparation of NCSC culture

Dorsal root ganglia from E11.5 day old EGFP mouse embryos were isolated and used to generate NCSC neurospheres (NL38 cell line) from the so-called boundary cap [16], [17]. Briefly, mice were anesthetized by intraperitoneal injection of xylazine (RompunVR vet.; http://www.bayer.com) and ketamine (KetaminolVR vet.; http://www.intervet.com) (10 and 100 ng per gram body weight, respectively) and the uterus was removed from the anaesthetised pregnant mouse and placed in cold PBS. Embryos were separated and rinsed in PBS, placed in DMEM/F12 medium (Invitrogen, Carlsbad, CA, USA), supplemented with N-2 (Invitrogen, Carlsbad, CA, USA) (N-2 medium), after which the dorsal root ganglia were removed and collected. Collected dorsal root ganglia were allowed to settle before removing the supernatant fractions and adding a collagenase/dispase (1 mg/mL) (Roche Diagnostics Scandinavia, Bromma, Sweden) and DNase solution (0.5 mg/mL) (Sigma-Aldrich) in N-2 medium and incubating for 20 to 30 min in a 37°C water bath. This was followed by rinsing in N-2 medium supplemented with B-27 (1∶50) (Invitrogen, Carlsbad, CA, USA) titurating and plating of 1 to 2×10^5^ cells/well in a 24-well dish after dissociation. Cells were placed directly into 500 µL N-2 medium containing B-27, epidermal growth factor (20 ng/mL) (R&D Systems, Minneapolis MN, USA) and basic fibroblast growth factor (20 ng/mL) (R&D Systems). After 12 h, non-adherent cells were removed together with half of the medium before adding 250 µL of fresh medium. The medium was then changed every other day (50% of the medium replaced with fresh medium) before neurospheres began to form. Neurospheres from passages 4 to 5 were trypsinised for 5 min to generate free single cell suspensions for subsequent co-culture with beta-TC6 cells.

### *In vitro* treatment of cells

10^5^ dispersed NCSCs were plated in 24-well plates or 0.4 mm pore size PET track-etched membrane inserts (Falcon) and were allowed to cover most of the surface during three days of culture in the N-2 culture medium given above. All wells/inserts were pre-coated with laminin (10 µg/mL) to promote efficient spreading of the NCSCs. After three days 10^4^ beta-TC6 cells were plated either alone or together with the NCSC cells. At this stage the culture medium was changed to RPMI-1640 medium containing the same supplements as given above. For co-culture with inserts the beta-TC6 cells were plated so that the cells were located in the bottom of the well and the NCSC cells were above in the inserts. After two days of co-culture, cells were either left untreated or treated with a mixture of cytokines (20 ng/mL IL-1β+20 ng/mL IFN-γ; Peprotech) for an additional 48 hours. After the cytokine exposure period culture medium samples were analysed for nitrite content using the Griess reagent [4].

### Flow cytometry analysis of cell viability

*In vitro* cultures of beta-TC6 cells, NCSCs or beta-TC6 + NCSCs were labelled for 10 min at 37°C with 10 µg/mL of propidium iodide (Sigma-Aldrich). In some experiments cells were treated with the gap junction inhibitor carbenoxolone (50 μM; Sigma Aldrich) during the 48 h cytokine exposure period. The cells were washed once with PBS and then trypsinised for 5 min at 37°C. Cell suspensions were analysed in a Becton Dickinson FACSCalibur flow cytometer for FL1 (GFP) and FL3 (propidium iodide) fluorescence. Cell death frequencies were quantified for GFP positive and GFP negative cells separately, and expressed as percentage of total GFP positive and negative cell numbers, respectively.

### Immunostaining

Cells were fixed in 4% buffered paraformaldehyde at room temperature for 5 minutes then washed with PBS prior to permeabilisation and blocking using PBS with 0.1% triton® X-100 (Sigma), 1% BSA (Sigma), and 3% fetal calf serum. The cells were incubated with primary antibodies in PBS with 1% BSA and 1% fetal calf serum for 30 minutes at 37°C before washing two times with PBS. The cultures were then incubated with secondary antibodies for 30 minutes at 37°C and rinsed three times in PBS for 15 minutes, the second wash included Hoechst 33242 (11 ng/mL, Invitrogen). Coverslips were mounted on glass slides with Dako Cytomation fluorescent mounting solution. Primary antibodies were as follows: anti-NOS2 (monoclonal mouse, 1∶100, Santa Cruz), anti-beta catenin (polyclonal rabbit, 1∶100, Abcam), anti-pan cadherin (monoclonal mouse, 1∶100, Abcam), PE-conjugated alpha6-integrin (1∶100, Abcam), anti-laminin (polyclonal rabbit, 1∶200, Sigma), and phospho-FAK (pY397) (polyclonal rabbit, 1∶100, Invitrogen). Secondary antibodies were Cy3 (donkey anti-mouse, 1∶500, Jackson laboratories), and Alexa flour 555 (goat anti-rabbit, 1∶600, Invitrogen).

### Immunoblotting

Cells were lysed in SDS sample buffer, boiled for 5 min and separated by SDS-PAGE. Proteins were electrophoretically transferred onto a Hybond-P membrane (GE Healthcare, Uppsala, Sweden). Membranes were incubated with the following primary antibodies: mouse anti-NOS2 (C-11, Santa Cruz) and mouse anti-tubulin (Santa Cruz). The immunodetection was performed as described for the ECL immunoblotting detection system (GE healthcare) and using the Kodak Image station 4000 MM.

### Microscopic analysis

Immunolabelled slides were analysed in a Nikon Eclipse E800 fluorescence microscope.

### Flow cytometry analysis of ERK, Akt and FAK phosphorylation

The following primary antibodies were used: PerCP-conjugated Phospho-ERK1/2(T202/Y204) (Becton Dickinson), PE-conjugated Phospho-Akt(T473) (Becton Dickinson), rabbit Phospho-FAK(Y397) (Invitrogen) and rabbit Phospho-FAK(Y576/577) (Cell Signaling). Secondary antibody for the phospho-FAK antibodies was DyLight 650 (goat anti-rabbit, Abcam). Briefly, cells were rapidly fixated, according to the protocol of the BD Phosflow kit (Becton-Dickinson). The cells were then permabilised, incubated with antibodies and washed for flow cytometry analysis (FACSCalibur) of fluorescent signals.

### Statistical analysis

Data were analysed using Student's paired t-test. Significance was taken at p<0.05.

## Results

### Effects of NCSC-beta-TC6 co-culture on cytokine-induced cell death, nitrite production and iNOS expression

**Figure 1:**
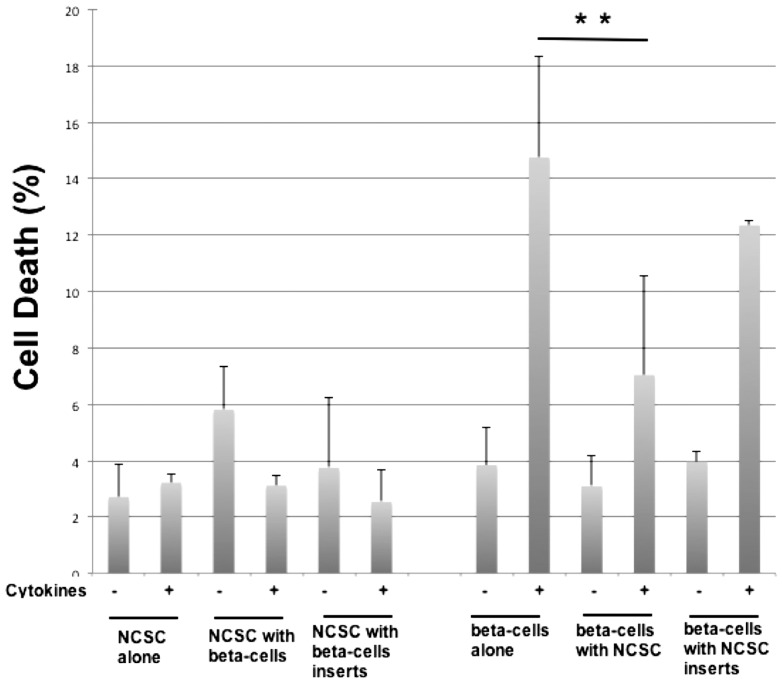
Co-culture of beta-TC6 cells with NCSCs in direct contact results in protection against cytokine-induced beta-cell death. NCSCs were plated on laminin-coated plates or inserts and after three days beta-TC6 cells were added. After another 48 hours, IL-1β (20 ng/mL) and IFN-β (20 ng/mL) was added. 48 hours later cells were labeled with propidium iodide, trypsinised and analysed for cell death by flow cytometry. GFP-positive cells (NCSCs) and GFP-negative cells (beta-TC6) were gated and analysed separately. Results are means ± SEM for 5 independent experiments. ** denotes p<0.01 using Student's paired t-test.

In a previous study we observed that NCSCs partially protect beta-cells against cytokine-induced death when co-cultured [14]. However, we could not determine whether protection was obtained by the release of soluble factors, or whether a direct cell-cell contact was required. We have therefore presently co-cultured NCSC with insulin producing mouse beta-TC6 cells with or without 0.4 micrometer cell culture inserts that allow passage of soluble factors, but not direct cell-to-cell contact. We observed that cytokine-induced beta-TC6 cell death was counteracted by co-culture with NCSC (Figure 1). When cells were separated by inserts that allow passage of soluble factors no protection against cytokines was detected (Figure 1). This suggests that a direct contact between beta-TC6 cells and NCSCs was needed for protection. NCSCs did not respond to cytokine exposure, either when cultured alone or in combination with beta-TC6 cells (Figure 1).

**Figure 2:**
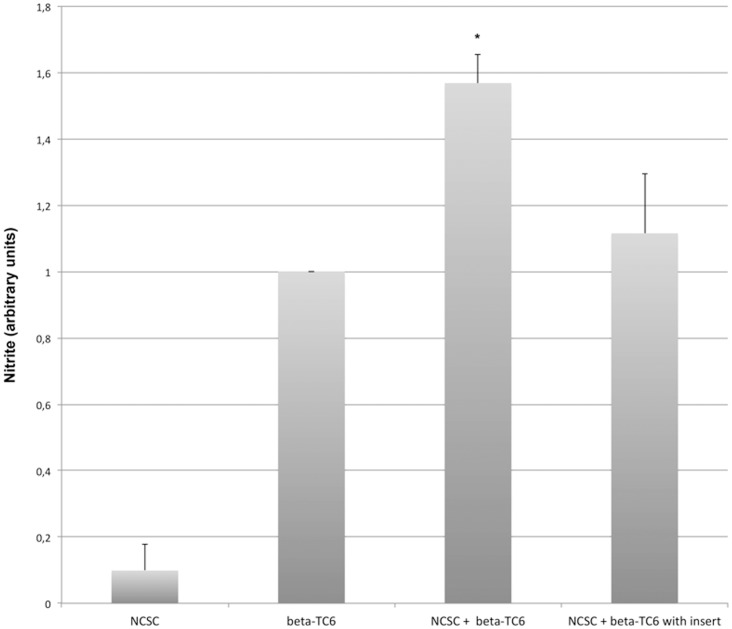
Co-culture of beta-TC6 cells with NCSCs in direct contact results in increased nitrite production in response to cytokine exposure. NCSCs were plated on laminin-coated plates or inserts and after three days beta-TC6 cells were added. After 48 hours of co-culture IL-1β (20 ng/mL) and IFN-γ (20 ng/mL) was added during another 48 hour culture period. At the end of this culture period culture medium samples were analysed for nitrite using the Griess reagent. Results are means ± SEM for 4 independent experiments. * denotes p<0.05 using Student's paired t-test.

Cytokines promote rodent beta-cell death *in vitro* mainly by activation of the inducible nitric oxide synthase (iNOS) enzyme [1]. To investigate whether co-culture of beta-TC6 cells with NCSCs affected iNOS induction, we next analysed nitrite, which is the end product of iNOS-derived nitric oxide. Nitrite levels in cells not exposed to the cytokines IL-1β and IFN-γ were non-detectable (results not shown). In cytokine-exposed cells nitrite production was some 10-fold higher in beta-TC6 cells than in NCSCs when cultured alone (Figure 2). Co-culture of beta-TC6 cells and NCSCs separated by an insert did not affect nitrite production (Figure 2). However, co-culture without inserts resulted in a potentiated nitrite production as compared to beta-TC6 cells alone (Figure 2).

**Figure 3:**
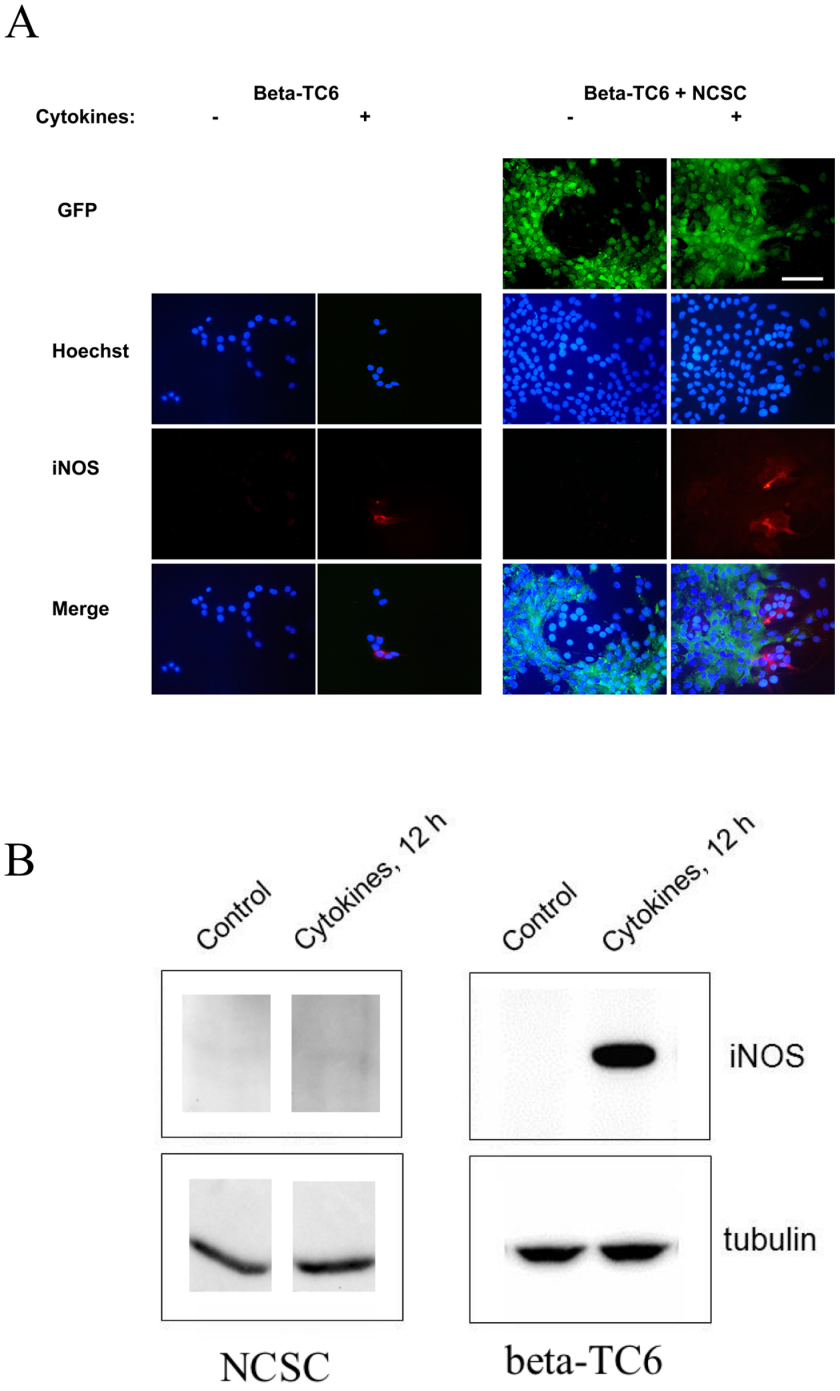
Expression of iNOS can be detected in beta-TC6 cells. (A) Non-GFP positive beta-TC6 cells were either cultured alone (left panels) or together with GFP-positive NCSC cells (right panels, green cells) on laminin-coated cover slips. Cells were treated with 20 ng/mL of IL-1β and 20 ng/mL of IFN-γ for 48 hours. Cells were then fixated, permeabilized and stained for iNOS (red). Nuclei were visualized by Hoechst. Fluorescence was then photographed in a fluorescence microscope using a 40× lens. The white scale bar depicts a distance of 100 µm. Results are representative for three independent experiments. (B) Immunoblot analysis of NCSCs (left panel) and beta-TC6 cells (right panel). Both cell types were cultured alone. Cells were stimulated for 12 hours with IL-1β and IFN-γ and then analysed for iNOS expression by immunoblot analysis. As loading control tubulin was used. The results are representative for three observations.

To evaluate the origin of cytokine-induced nitric oxide production, we stained the cells cultured without inserts for iNOS immunoreactivity. The beta-TC6 cells were observed as non-GFP positive cells located as cell islands surrounded by the GFP-positive NCSC monolayer (Figure 3A). No iNOS staining could be observed in cells not treated with cytokines. Cytokine treatment resulted in a clear loss of beta-TC6 cells when cultured alone (Figure 3A). Among the remaining beta-TC6 cells some exhibited clear iNOS immunoreactivity. Also when co-cultured with NCSCs some beta-TC6 cells were iNOS positive (Figure 3A). None of the NCSCs displayed iNOS immunoreactivity. To further corroborate that iNOS is expressed only in cytokine-treated beta-TC6 cells, and not in NCSCs, we performed immunoblot analysis of the two cells types. We observed iNOS immunoreactivity in cytokine-treated beta-TC6 cells, but not in NCSCs (Figure 3B). Thus, nitric oxide is mainly produced by beta-TC6 cells, and a higher survival rate, induced by direct co-culture with NCSCs, allows a further enhanced and/or prolonged nitric oxide production.

### Effects of beta-TC6 and NCSC cell co-culture on laminin, alpha6-integrin and phospho-FAK immunofluorescence

**Figure 4:**
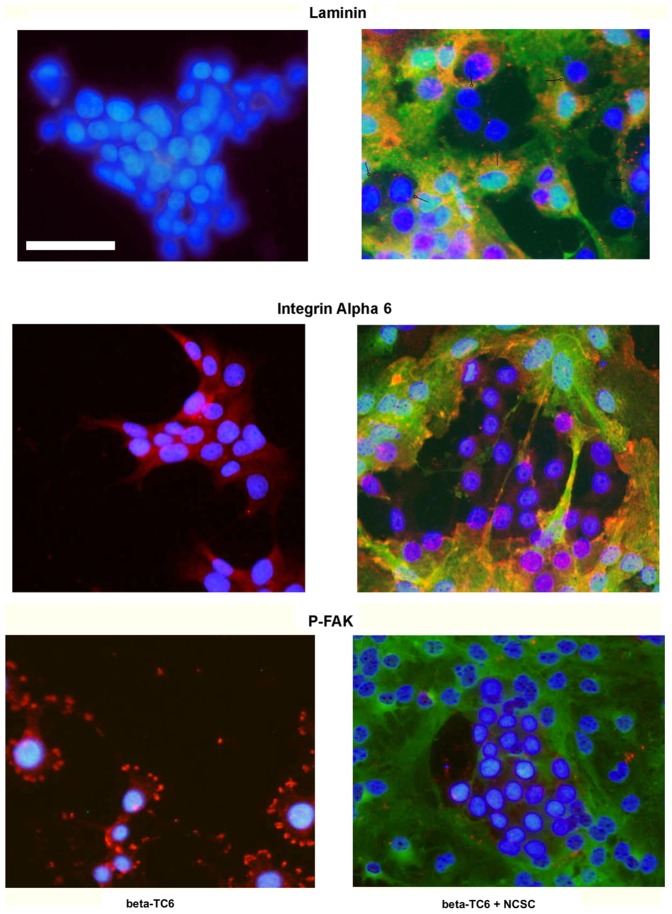
Co-culture of beta-TC6 cells with NCSCs affects beta-TC6 cell focal adhesions. Non-GFP positive beta-TC6 cells were either cultured alone (left panels) or together with GFP-positive NCSC cells (right panels, green cells) on laminin-coated cover slips. Cells were treated with 20 ng/mL of IL-1β and 20 ng/mL of IFN-γ for 48 hours and were then fixated, permeabilized and stained for laminin (orange), integrin alpha VI (orange) or phospho-FAK(Y397) (red). Nuclei were visualized by Hoechst. Fluorescence was photographed in a fluorescence microscope using a 20× lens. Circleheads indicate positions of NCSC laminin accumulations close to beta-TC6 cells. The scale bar depicts a distance of 50 µm. Results are representative for three independent experiments.

To explore the mechanisms by which direct contacts between NCSCs and beta-TC6 cells promote improved beta-cell survival, we next immunostained for the laminin-integrin-focal adhesion kinase (FAK) cell adhesion pathway. No laminin immunoreactivity was observed in beta-TC6 cells cultured alone (Figure 4). The staining of the co-cultured cells revealed that NCSCs, but not beta-TC6 cells, produce laminin (Figure 4). The laminin immunoreactivity of the NCSCs often came in close contact with the laminin negative beta-cells (Figure 4, circleheads). This raises the possibility that beta-TC6 cells, via integrins, attach to the NCSC-produced extracellular matrix protein laminin. We therefore analysed alpha6-integrin immunostaining, a laminin-binding extracellular matrix receptor [18]. Beta-TC6 cells cultured alone displayed a weak cytoplasmic alpha6-integrin signal (Figure 4). When co-cultured with NCSCs, however, this signal was not increased. Instead, NCSCs displayed clear alpha6-integrin signals, which were often localized to plasma membrane regions of NCSCs (Figure 4). This indicates that laminin/alpha6-integrin interactions occur predominantly in NCSCs, but may also be present in beta-TC6 cells. To test this further, we next studied phospho-FAK(Y397) immunostaining. Integrin receptor activation affects beta-cell function, survival and proliferation [19], and often stimulates FAK phosphorylation and accumulation at focal adhesion sites. Beta-TC6 cells cultured in the absence of NCSCs contained numerous phospho-FAK stained focal adhesions localized at the plasma membrane (Figure 4). These phospho-FAK positive sites were less frequent in NCSCs. Interestingly, beta-TC6 cells, when co-cultured with and surrounded by NCSCs, displayed a clear decrease in the number of phospho-FAK focal adhesions (Figure 4). Thus, direct contact with NCSCs may efficiently hinder the formation of beta-TC6 cell focal adhesions, even though laminin is produced by surrounding NCSCs.

Cytokine exposure did not affect laminin or alpha6-integrin immunostaining patterns in beta-TC6 cells, neither when cultured alone nor when co-cultured with NCSCs (results not shown). However, the phospho-FAK(Y397) signal appeared stronger in cytokine-exposed beta-TC6 cells as compared to non-cytokine exposed beta-TC6 cells (results not shown).

### Effects of beta-TC6 and NCSC cell co-culture on the phosphorylation of ERK, Akt and FAK

**Figure 5:**
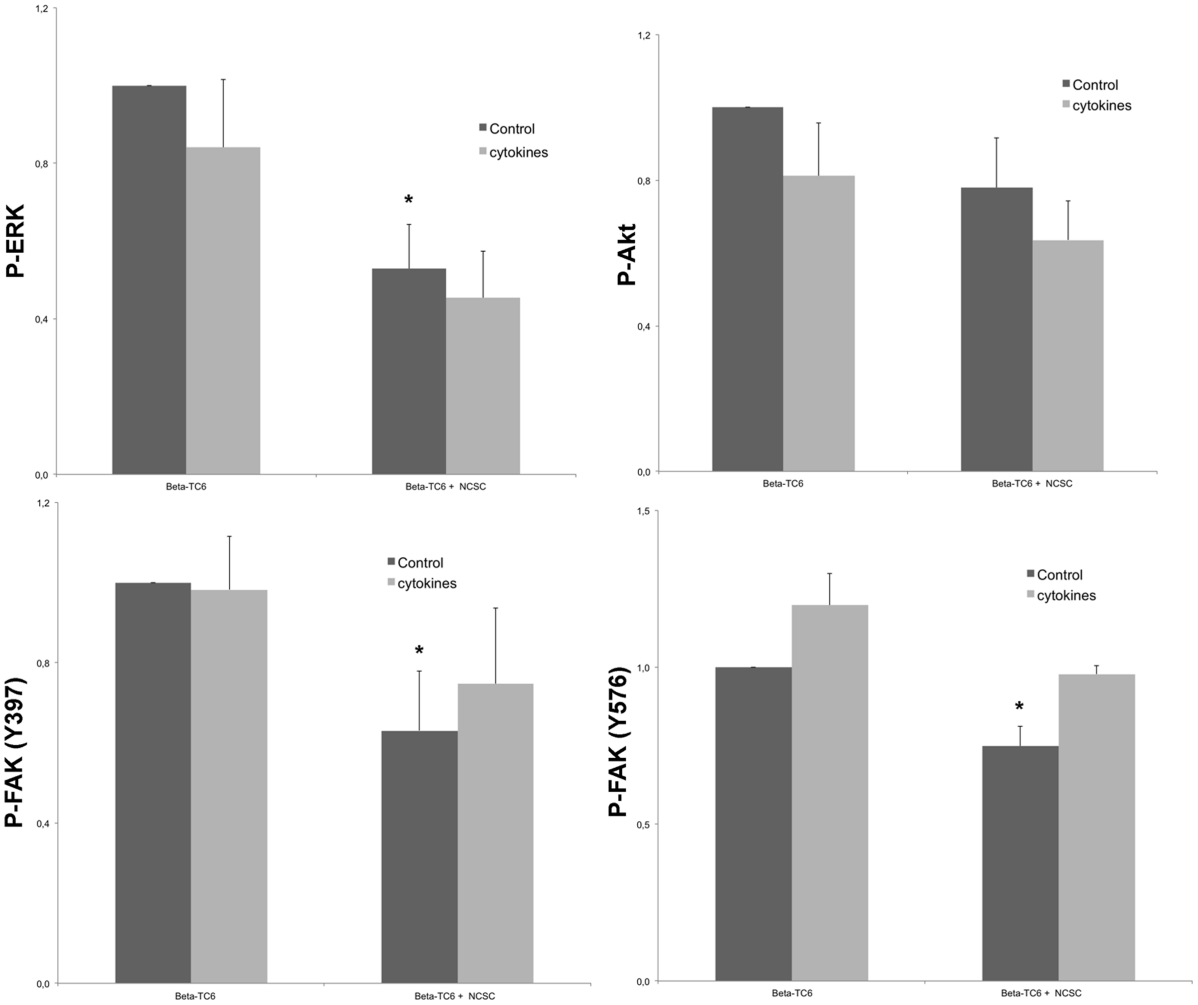
Effects of co-culture on beta-TC6 cell ERK, AKT and FAK phosphorylation. NCSCs were plated on laminin-coated plates or inserts and after three days beta-TC6 cells were added. After 48 hours of co-culture IL-1β and IFN-γ was added during another 48 hour culture period. Cells were then analysed for ERK1/2(T202/Y204), Akt(T473), FAK(Y397) and FAK(Y576/577) phosphorylation using the Becton Dickinson Phosflow technique. Results are means ± SEM for 3 independent observations. * denotes p<0.05 using Student's paired t-test.

To further corroborate our finding that direct contacts with NCSCs dampen beta-TC6 cell FAK activation, we next analysed the effect of co-culture on beta-TC6 cell ERK(T202/Y204), Akt(S473), FAK(Y397) and FAK(Y576/577) phosphorylation by flow cytometry and the Becton-Dickinson Phosflow technique. This approach allows quantification of phospho-protein levels in a specific cell population, rather than in all cells of the co-culture. Intracellular ERK, Akt and FAK phosphorylation was studied in beta-TC6 and NCSCs, by FL1-gating of non-GFP and GFP-positive cells separately, and then quantifying FL2 (PE-conjugated phospho-Akt antibody) and FL3 (PerCP-conjugated phospho-ERK) signals in the two cell populations. We observed significant decreases in phospho-ERK, phospho-FAK(Y397) and phospho-FAK(Y576/577) levels in beta-TC6 cells co-cultured with NCSCs, as compared to beta-TC6 cells cultured alone (Figure 5). There was also a trend to lower phospho-Akt, but this did not reach statistical significance. Nevertheless, the present flow cytometry results correspond well to the immunostaining results (Figure 4), as both methods report lowered FAK activation.

Exposure to cytokines for 48 hours did not significantly affect ERK and Akt phosphorylation, but the phosphorylation of FAK(Y576) was stimulated by cytokines in beta-TC6 cells (Figure 5). Phosphorylation levels were not affected in NCSCs, either cultured alone or co-cultured with beta-TC6 cells (results not shown).

### Effects of beta-TC6 and NCSC cell co-culture on cadherin and beta-catenin immunofluorescence

**Figure 6:**
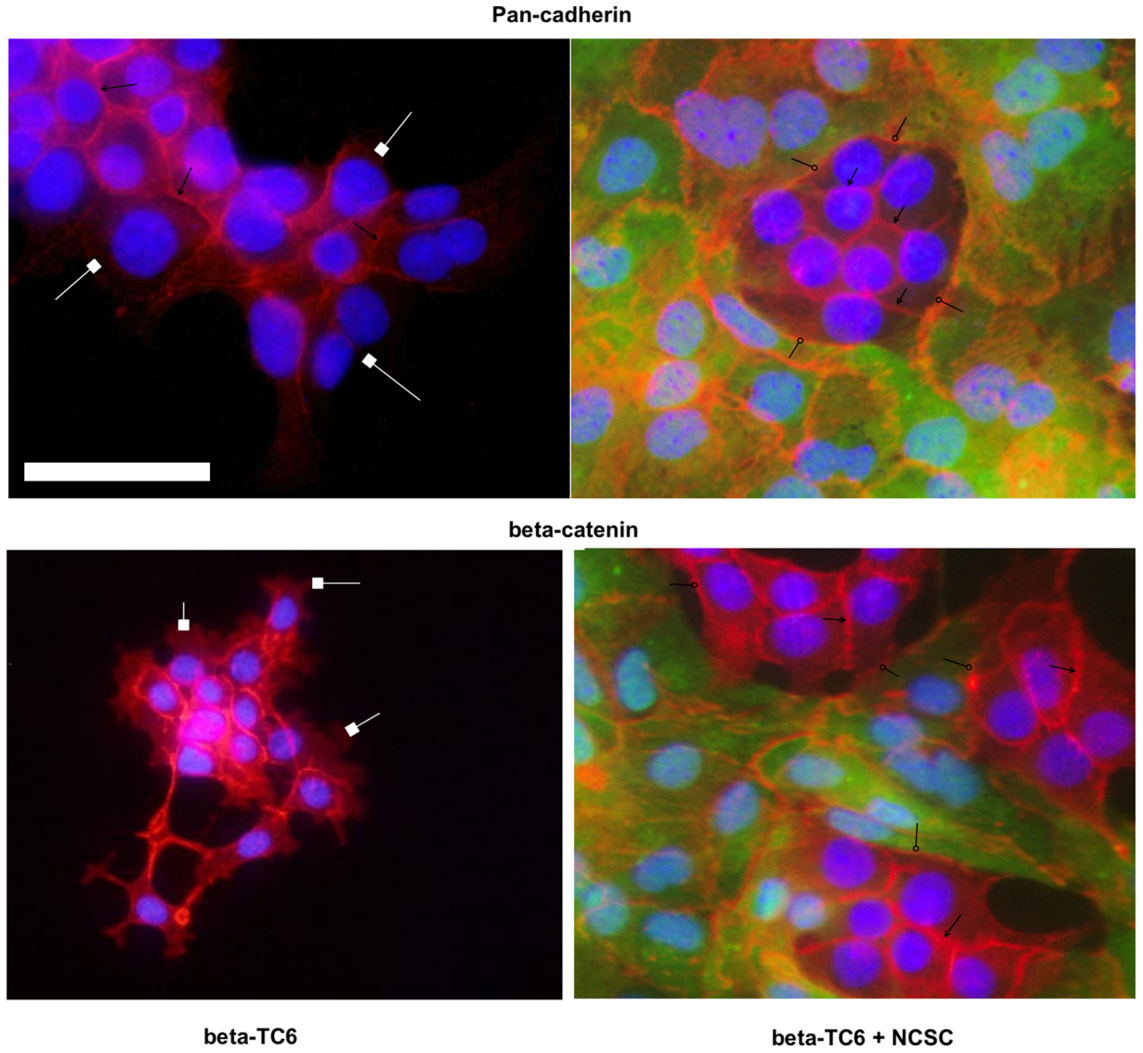
Beta-TC6 cells and NCSCs form cadherin junctions. Non-GFP positive beta-TC6 cells were either cultured alone (left panels) or together with GFP-positive NCSC cells (right panels, green cells) on laminin-coated cover slips. Cells were then fixated, permeabilized and stained for pan-cadherin or beta-catenin (red). Nuclei were visualized by Hoechst. Fluorescence was then photographed in a fluorescence microscope using a 20× lens. Arrowheads indicate positions of cadherin/beta-catenin junctions between beta-TC6 cells and circleheads indicate positions of junctions between beta-TC6 cells and NCSCs. Squareheads indicate positions of outer circumference of beta-TC6 not surrounded by NCSCs. The scale bar depicts a distance of 5 µm. Results are representative for three independent experiments.

Cadherin junctions are strong mediators of beta-cell survival [20] and NCSC competence [21], [22]. Using a pan-cadherin antibody, we next analysed the microscopic appearance of cadherin junctions in the beta-TC6/NCSC co-cultures. We registered cadherin accumulation at the plasma membrane of adjacent cells, both between beta-TC6 cells (Figure 6, arrowheads) and between NCSCs, as well as between beta-TC6 cells and NCSCs (Figure 6, circleheads). Also beta-catenin, the intracellular link between cadherin junctions and the cell cytoskeleton, displayed a similar staining pattern (Figure 6). We observed that peripheral beta-TC6 cells, which were not completely surrounded by other cells, at the circumference of the cell monolayer lacked cadherin and beta-catenin accumulations (Figure 6, squareheads). In contrast, both cadherin and catenin were abundantly present in beta-TC6 cells when the cells were tightly surrounded by NCSCs (Fig. 6, circleheads). This indicates that beta-TC6 cells form functional cadherin junctions with NCSCs that fill out the entire circumference of the cells. Beta-catenin and cadherin immunofluorescence patterns in beta-TC6 and NCSC cells were not affected by cytokines (results not shown).

### Inhibition of gap junction activity did not affect co-culture-induced protection against cytokine-induced beta-TC6 cell death

**Figure 7:**
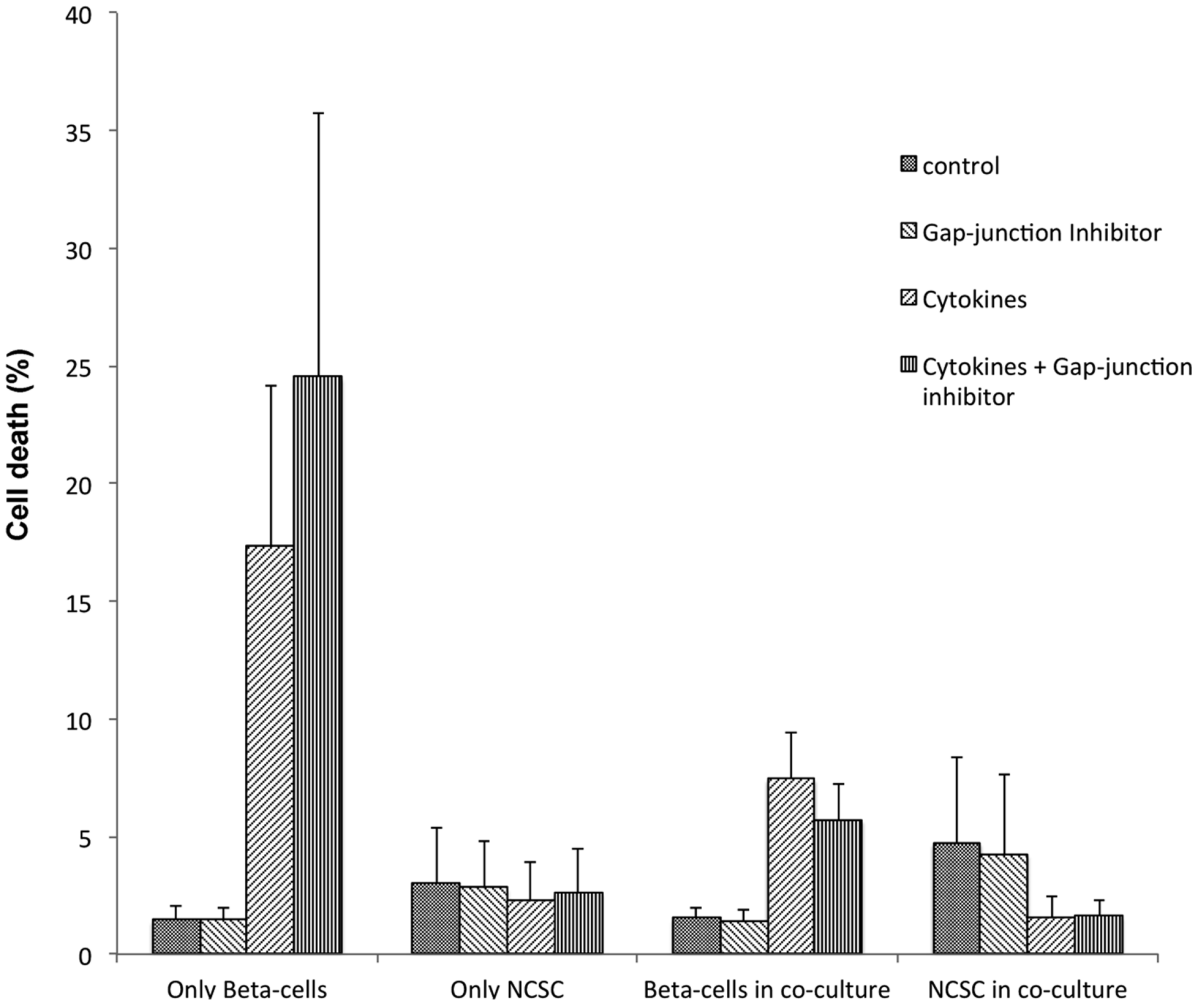
A gap junction inhibitor does not affect co-culture-induced protection against cytokines. NCSCs were plated on laminin coated plates and after three days beta-TC6 cells were added. After another 48 hours, IL-1β (20 ng/mL) and IFN-γ (20 ng/mL) was added alone or in combination with the gap junction inhibitor carbenoxolone (50 µM). 48 hours later cells were labeled with propidium iodide, trypsinised and analysed for cell death by flow cytometry. GFP-positive cells (NCSCs) and GFP-negative cells were gated and analysed separately. Results are means ± SEM for three separate observations.

As gap junctions have been reported to promote beta-cell survival [23], we performed cell viability determinations on beta-TC6 cell/NCSC co-cultures with the gap junction inhibitor carbenoxolone. We observed that cytokine-induced beta-TC6 cell death was not affected by the gap junction inhibitor, neither when beta-TC6 cells were cultured alone, nor when beta-TC6 cells were co-cultured with NCSCs (Figure 7).

## Discussion

Previous studies have reported stimulatory effects of the co-culture of NCSCs and beta-cells on beta-cell proliferation [15] and beta-cell function following transplantation to diabetic mice [13]. In addition, it has also been reported that beta-cell survival in response to cytokines is increased when co-cultured with NCSCs [14]. Although the beneficial effect of co-culture on beta-cell proliferation required direct cell-to-cell contacts [15], it has remained unknown whether protection against cytokine-induced death was mediated by soluble factors or mediated by direct cell-to-cell contact. However, in the present investigation we observe that also improved viability following cytokine exposure requires direct cell-to-cell contacts. Thus, the same cell-to-cell contacts might mediate not only increased beta-cell proliferation, but also improved beta-cell function and survival in response to cytokines.

The nature of the direct cell-to-cell contacts has previously not been elucidated. Here we analysed cadherin junctions, integrin-ECM interactions and gap junctions between NCSCs, islet cells and these cells in co-cultures. We report that beta-TC6 and NCSC cells are linked by cadherin junctions. These structures are likely to be composed of Neuronal-cadherin (N-cadherin) and/or Epithelial-cadherin (E-cadherin), as both beta-cells and NCSCs are known to express these homophilic adhesion molecules, depending on the developmental stage of the two cell types [24]–[26]. Cadherin junctions between beta-cells promote improved survival and function [20], and it is possible that also cadherin junctions between NCSC and beta-TC6 cells exert similar effects. The cadherin-induced signaling pathways that mediate resistance to cell death are probably highly complex and context dependent, but studies have reported increased calcium inflow [27], decreased beta-catenin nuclear translocation [28] and decreased ERK phosphorylation [29] as possible down-stream signaling events.

In addition to cadherin junctions, we presently also observed that direct beta-TC6/NCSC interactions result in increased laminin production and decreased ERK/FAK signaling. It is not clear why the presence of NCSCs promoted decreased ERK/FAK signaling, but we can envisage two possible explanations. First, using pancreatic islet cells it has been reported that alpha6-integrin binding to collagen IV stimulates ERK activation, whereas integrin binding to laminin instead down-regulates ERK activation via a lowering of fibroblast growth factor receptor-1 (FGFR1) signaling [18]. In addition, insulin producing INS-1 cells responded to collagen IV, but not to laminin, with a stimulated FAK phosphorylation [30]. Therefore, the NCSC-derived laminin may have substantially altered the composition of the extra-cellular matrix so that the collagen effects fade out and the FAK/ERK-phosphorylation is attenuated. Second, it is also possible that strong cadherin junctions between beta-TC6 cells and NCSCs, via cis and trans lateral interactions, physically compete out beta-TC6 cell focal adhesions leading to lowered FAK/ERK signaling. Strong cadherin junctions are associated with a maintained epithelial phenotype and resistance against epithelial-to-mesenchymal transition [31]. In addition, it has also been reported that cell interactions with specific laminins counteract epithelial to mesenchymal transition, thereby preventing de-differentiation and loss of beta-cell phenotype [32], [33]. Thus, both cadherin junctions and NCSCs-produced laminin might modulate beta-TC6 cell integrin signaling so that ERK and FAK signaling are down-regulated, epithelial-to-mesenchymal transition is inhibited and survival is preserved. It is tempting to speculate that the two alternative explanations, i.e. increased cadherin and increased laminin, act synergistically to preserve beta-cell function and survival. Indeed, laminin-activated integrins have been reported to stabilize E-cadherin junctions in other cell systems [34].

Gap junctions are also direct cell-to-cell contacts that are potential mediators of beta-cell survival [23]. In beta-cells it appears that connexin36 forms functional gap junctions that protect against cytokine-induced cell death [23]. On the other hand, it has been reported that NCSCs express connexin43 prior to differentiation into mature cell types [35]. As there are numerous examples of functional heteromeric gap junctions, it is possible that such are present also in the presently observed beta-TC6/NCSC junctions. However, we observed no effect of the gap junction inhibitor carbenoxolone on betaTC6 cell survival, indicating that putative beta-TC6/NCSC gap junctions do not mediate improved beta-TC6 cell survival.

In summary, given the well-established pro-survival role of cadherin junctions, it is likely that the presently observed direct cell-to-cell contacts participate in protection against cytokine-induced cell death. In order to further corroborate these findings, it appears at first highly motivated to block NCSC-to-beta-TC6 cadherin junctions using cadherin-disrupting antibodies [27]. Unfortunately, such a strategy would also disrupt beta-TC6-to-beta-TC6 junctions, which would probably make the interpretation of any putative results highly problematic. Therefore, other experimental approaches need to be developed for a better understanding of the direct NCSC/beta-TC6 cell contacts. Meanwhile, it is possible that islet transplantation trials would benefit from co-transplantation with NCSCs or from manipulations that promote beta-cell cadherin junctions with neighboring cells.

## References

[1] Eizirik DL, Mandrup-Poulsen T (2000) A choice of death-the signal- transduction of immune-mediated beta-cell apoptosis. Diabetologia 44: 2115–2133. 10.1007/s001250100021 11793013

[2] Suarez-Pinzon W, Sorensen O, Bleackley RC, Elliott JF, Rajotte RV, et al (1999) Beta-cell destruction in NOD mice correlates with Fas (CD95) expression on beta-cells and proinflammatory cytokine expression in islets. Diabetes 48: 21–28. 9892218 10.2337/diabetes.48.1.21

[3] Uno S, Imagawa A, Okita K, Sayama K, Moriwaki M, et al (2007) Macrophages and dendritic cells infiltrating islets with or without beta-cells produce tumour necrosis factor-alpha in patients with recent-onset type 1 diabetes. Diabetologia 50: 596–601. 17221211 10.1007/s00125-006-0569-9

[4] Welsh N (1996) Interleukin-1beta-induced ceramide and diacylglycerol generation may lead to activation of the c-Jun-NH2-terminal kinase and the transcription factor ATF2 in the insulin-producing cell line RINm5F. J Biol Chem 271: 8307–8312. 8626526 10.1074/jbc.271.14.8307

[5] Larsen CM, Wadt KA, Juhl LF, Andersen HU, Karlsen AE, et al (1998) Interleukin-1beta-induced rat pancreatic islet NO synthesis requires both the p38 and extracellular- signalregulated kinase 1/2 mitogen-activated protein kinases. J Biol Chem 273: 15294–15300. 9614146 10.1074/jbc.273.24.15294

[6] Flodström M, Welsh N, Eizirik DL (1996) Cytokines activate the nuclear factor kB (NF-kappaB) and induces nitric oxide production in human pancreatic islets. FEBS Lett 385: 4–6. 8641463 10.1016/0014-5793(96)00337-7

[7] Mokhtari D, Barbu A, Mehmeti I, Vercamer C, Welsh N (2009) Overexpression of the Nuclear Factor-{kappa}B subunit c-Rel protects against human islet cell death in vitro. Am J Physiol Endocrinol Metab 297: E1067–1077. 19706790 10.1152/ajpendo.00212.2009

[8] Ryan EA, Paty BW, Senior PA, Bigam D, Alfadhli E, et al (2005) Five-year follow-up after clinical islet transplantation. Diabetes 54: 2060–2069. 15983207 10.2337/diabetes.54.7.2060

[9] Davalli AM, Scaglia L, Zangen DH, Zangen DH, Hollister J, et al (1996) Vulnerability of islets in the immediate posttransplantation period. Dynamic changes in structure and function. Diabetes 45: 1161–1167. 8772716 10.2337/diab.45.9.1161

[10] Nekrep N, Wang J, Miyatsuka T, German MS (2008) Signals from the neural crest regulate beta-cell mass in the pancreas. Development 135: 2151–2160. 18506029 10.1242/dev.015859

[11] Kozlova EN, Jansson L (2005) In vitro interactions between insulin- producing beta cells and embryonic dorsal root ganglia. Pancreas 31: 380–384. 16258374 10.1097/01.mpa.0000181489.35022.4a

[12] Kozlova EN, Jansson L (2009) Differentiation and migration of neural crest stem cells are stimulated by pancreatic islets. Neuroreport 20: 833–838. 19421078 10.1097/WNR.0b013e32832b8e20

[13] Olerud J, Kanaykina N, Vasylovska S, King D, Sandberg M, et al (2009) Neural crest stem cells increase beta cell proliferation and improve islet function in co- transplanted murine pancreatic islets. Diabetologia 52: 2594–2601. 19823803 10.1007/s00125-009-1544-z

[14] Ngamjariyawat A, Turpaev K, Welsh N, Kokzlova EN (2012) Coculture of insulin-producing RIN5AH cells with neural crest stem cells protects partially against cytokine-induced cell death. Pancreas 41: 490–492. 22415669 10.1097/MPA.0b013e31823fcf2a

[15] Grouwels G, Vasylovska S, Olerud J, Leuckx G, Ngamjariyawat A, et al (2012) Differentiating neural crest stem cells induce proliferation of cultured rodent islet beta cells. Diabetologia. 55: 2016–25. 10.1007/s00125-012-2542-0 22618811

[16] Hjerling-Leffler J, Marmigere F, Heglind M, Cederberg A, Koltzenburg M, et al (2005) The boundary cap: a source of neural crest stem cells that generate multiple sensory neuron subtypes. Development 132: 2623–2632. 15872002 10.1242/dev.01852

[17] Aldskogius H, Berens C, Kanaykina N, Liakhovitskaia A, Medvinsky A, et al (2009) Regulation of boundary cap neural crest stem cell differentiation after transplantation. Stem Cells 27: 1592–1603. 19544468 10.1002/stem.77 PMC2733376

[18] Kilkenny DM, Rocheleau JV (2008) Fibrobast growth factor receptor-1 signaling in pancreatic islet b-cells is modulated by the extracellular matrix. Mol Endocinol 22: 196–205. 10.1210/me.2007-0241 PMC2194636 17916654

[19] Parnaud G, Hammer E, Rouiller DG, Armanet M, Halban PA, et al (2006) Blockade of b1 intergrin-laminin-5 interaction affects spreading and insulin secretion of rat b-cells attached on extracellular matrix. Diabetes 55: 1413–1420. 16644699 10.2337/db05-1388

[20] Parnaud G, Gonelle-Gispert C, Morel P, Giovannoni L, Muller YD, et al (2011) Cadherin engagement protects human b-cells from apoptosis. Endocrinology 152: 4601–4609. 21990317 10.1210/en.2011-1286

[21] Curchoe CL, Maurer J, McKeown SJ, Cattarossi G, Cimadamore F, et al (2010) Early Acquisition of Neural Crest Competence During hESCs Neuralization PLoS ONE. 5: e13890. 10.1371/journal.pone.0013890 PMC2976694 21085480

[22] Kléber M, Lee HY, Wurdak H, Buchstaller J, Riccomagno MM, et al (2005) Neural crest stem cell maintenance by combinatorial Wnt and BMP signaling. J Cell Biol 169: 309–320. 15837799 10.1083/jcb.200411095 PMC2171862

[23] Klee P, Allagnat F, Pontes H, Cerderroth M, Charollais A, et al (2011) Connexins protect mouse pancreatic b cells against apoptosis. J Clin Invest 121: 4870–4879. 22056383 10.1172/JCI40509 PMC3225984

[24] Dahl U, Sjödin A, Semb H (1996) Cadherins regulate aggregation of pancreatic beta-cells in vivo. Development 122: 2895–2902. 8787762 10.1242/dev.122.9.2895

[25] Moore R, Larue L (2004) Cell surface molecules and truncal neural crest ontogeny: A perspective. Birth Def Res 72: 140–150. 10.1002/bdrc.20014 15269888

[26] Johansson JK, Voss U, Kesavan G, Kostetskii I, Wierup N, et al (2010) N-cadherin is dispensable for pancreas development but required for beta-cell granule turnover. Genesis 48: 374–381. 20533404 10.1002/dvg.20628 PMC2921608

[27] Hauge-Evans AC, Squires PE, Persaud SJ, Jones PM (1999) Pancreatic beta-cell-to-beta-cell responses to nutrient stimuli: enhanced Ca2+ and insulin secretory resonses of MIN6 pseudoislets. Diabetes 48: 1402–1408. 10389845 10.2337/diabetes.48.7.1402

[28] Herzig M, Savarese F, Novatchkova M, Semb H, Christofori G (2007) Tumor progession induced by the loss of E-cadherin independently of beta-catenin/Tcf-mediated Wnt signaling. Oncogene 26: 2290–2298. 17043652 10.1038/sj.onc.1210029

[29] Iwashita J, Ose K, Ito H, Murata J, Abe T (2011) Inhibition of E-cadherin dependent cell-cell contact promotes MUC5AC mucin production through the activation of epidermal growth factor receptors. Biosci Biotechnol Biochem 75: 688–693. 21512244 10.1271/bbb.100830

[30] Krishnamurthy M, Li J, Al-Masiri M, Wang R (2008) Expression and function of alphabeta1 integrins in pancreatic beta (INS-1) cells. J Cell Commun Signal 2: 67–79. 19023675 10.1007/s12079-008-0030-6 PMC2648043

[31] Heuberger J, Birchmeier W (2010) Interplay of cadherin-mediated cell adhesion and canonical Wnt signaling. Cold Spring Harb Perspect Biol 2: a002915. 20182623 10.1101/cshperspect.a002915 PMC2828280

[32] Chapman HA (2012) Epithelial responses to lung injury: role of the extracellular matrix. Proc Am Thorac Soc 9: 89–95. 22802280 10.1513/pats.201112-053AW PMC5830703

[33] Banerjee M, Virtanen I, Palgli J, Korsgren O, Otonkoski T (2012) Proliferation and plasticity of human beta cells on physiologically occurring laminin isoforms. Mol Cell Endocrinol 355: 78–86. 22314207 10.1016/j.mce.2012.01.020

[34] Stipp CS (2010) Laminin-binding integrins and their tetraspanin partners as potential antimetastatic targets. Exp Rev Mol Med 12 doi:10.1017/S1462399409001355. 10.1017/S1462399409001355 PMC2811424 20078909

[35] Li J, Habbes HW, Eiberger J, Willecke K, Dermietzel R, et al (2006) Analysis of connexin expression during mouse schwann cell development identifies connexin29 as a novel marker for the transition of neural crest to precursor cells. GLIA 55: 93–103. 10.1002/glia.20427 17024657

